# Total aortic arch replacement surgery with a Core temperature of 34 °C

**DOI:** 10.1186/s13019-019-1001-0

**Published:** 2019-11-04

**Authors:** Quan Li, Hong Qu, Tianqi Liu, Jianmin Yu, Meng Lv

**Affiliations:** 1 Division of Cardiac Surgery, 16766 Jingshi Road, Jinan, 250014 Shandong China; 2 Division of Anaesthesiology, Shandong Provincial Qianfoshan Hospital, the First Hospital Affiliated with Shandong First Medical University, Affiliated Hospital of Shandong University, Jinan, Shandong China

**Keywords:** Acute type a aortic dissection, Frozen elephant trunk procedure, Total aortic arch replacement

## Abstract

**Background:**

Traditional aortic arch replacement surgery must be performed under moderate or deep hypothermia (22–28 °C) and circulatory arrest. Hypothermia and hypoperfusion can cause damage to the nervous system; therefore, postoperative brain and spinal cord complications are common. Improvements in surgical techniques are necessary to solve this problem. Herein, we report a method of total aortic arch replacement that can be performed at a core temperature of 34 °C, similar to other simple cardiac operations.

**Case presentation:**

Four patients underwent surgery with this technique (3 males and 1 female, aged 48 to 67 years). Computed tomography angiography performed at admission showed a total aortic dissection, resulting in a diagnosis of Stanford type A aortic dissection. The patients underwent emergency aortic sinus remodelling, ascending aortic replacement, modified aortic arch replacement, and elephant trunk stenting. No patients had neurological complications. During a follow-up of more than 1-month, no patients had aortic valve regurgitation or anastomotic leak.

**Conclusions:**

This technique can increase the operating temperature by approximately 6 to 12 °C and reduce the circulatory arrest time by approximately 18 to 28 min. All of the patients recovered well without any neurological complications, demonstrating the feasibility and safety of this technique. We believe that this technique can serve as a good alternative strategy for managing aortic dissection and aneurysm, especially for young surgeons who are acquiring experience in arch replacement surgery.

## Background

Currently, aortic arch replacement surgery is typically performed under moderate or deep hypothermia and circulatory arrest because the open anastomosis at the distal end of the arch needs to be performed without blood supply to the spinal cord and abdominal organs [1–3]. To improve the body’s tolerance to ischaemia, continuous cooling is necessary. Under moderate or deep hypothermic conditions (28 °C to 25 °C), the safe time limit for spinal cord ischaemia is approximately 20 to 30 min, requiring the surgeon to be experienced and skilled in anastomosis techniques to minimize ischaemic time. However, the damage to the body caused by hypothermia and hypoperfusion is unavoidable [2–4]. Herein, we report an aortic arch replacement procedure that does not require moderate or deep hypothermia and allows almost continuous perfusion. This technique can increase the operating temperature by approximately 6 to 12 °C and reduce the circulatory arrest time by approximately 18 to 28 min.

## Case presentation

Four patients underwent surgery with this technique (3 males and 1 female, aged 48 to 67 years). All patients had hypertension, and the female patient had chronic obstructive pulmonary disease (COPD). All patients were admitted to the hospital due to chest pain within 1 day. Computed tomography angiography (CTA) performed at admission showed a total aortic dissection, resulting in a diagnosis of Stanford type A aortic dissection. Cardiac colour ultrasonography revealed different levels of pericardial haemorrhage, right and noncoronary sinus dissection and intimal floating at 2 to 4 cm above the sinus tube junction. Two patients showed mild to moderate aortic valve regurgitation. The patients underwent emergency aortic sinus remodelling, ascending aortic replacement, aortic arch replacement, and elephant trunk stenting.

**Fig. 1:**
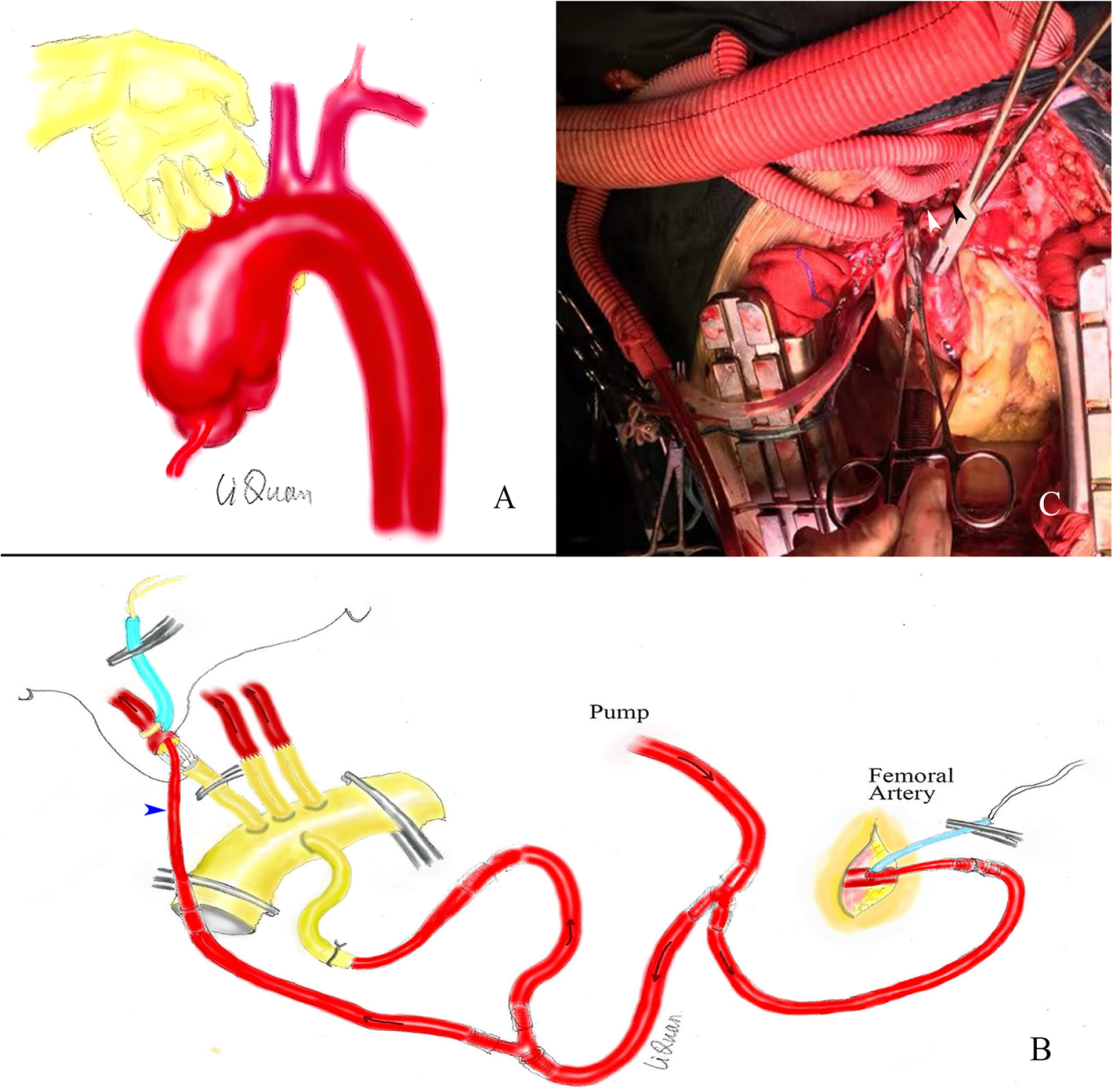
**a** Method for separation of the aortic arch and tracheal space. **b** Method of cannulation for extracorporeal circulation and perfusion. **c** “Branch-first” technique and myocardial protection solution infusion. Closed left common carotid artery stump (black arrow); Retained right brachiocephalic artery stump (white arrow)

An anterior medial incision was created to separate the three branches of the aorta and carefully dissociate the aortic arch and tracheal space with the left index finger (Fig. 1a). This gap was loose, and the aortic arch and tracheal space were gently separated to prevent rupture of the aneurysm. The separation was limited to the right posterior arch of the left common carotid artery to prevent damage to the left recurrent laryngeal nerve. The space was used as the clamping site of the middle arch. We converted open anastomosis at the distal end of the arch into closed anastomosis with continuous perfusion. Cannulation was performed via the femoral artery and the fourth branch of the artificial four-branch vessel (InterVascular SAS, La Ciotat, France) for perfusion (Fig. 1b). A two-stage venous cannula was placed in the right atrium for extracorporeal circulation. Before starting extracorporeal circulation, the artificial four-branched blood vessels were pre-filled with CO_2_, and the gas in the duct (including the four branch vessels) was removed by blood return from the femoral artery cannula.

First, using the “branch-first” technique, the three branches of the aorta were anastomosed (Fig. 1c). At ambient temperature, the left subclavian artery was anastomosed without extracorporeal circulation. Immediately after the anastomosis, extracorporeal circulation and slow cooling were initiated. The perfusion flow of the extracorporeal circulation was 900–1300 ml/min. The core temperature was maintained at ≥34 °C for the entire period of extracorporeal circulation. The same method was used to anastomose the left common carotid artery. When the right brachiocephalic artery was anastomosed, a 12–14 F arterial cannula and perfusion were required for brain protection (Fig. 1b blue arrow). The left subclavian and left common carotid artery stumps were sutured closed, and the right brachiocephalic artery stump was retained (Fig. 1c). The fourth patient underwent another perfusion procedure. After the left subclavian artery anastomosis, extracorporeal circulation was not performed immediately. The left subclavian artery was perfused by blood return from the femoral artery cannula. After the anastomosis of all three branches, the two-stage venous cannula was performed and extracorporeal circulation began. This method can reduce cardiopulmonary bypass time by about 30 min.

**Fig. 2:**
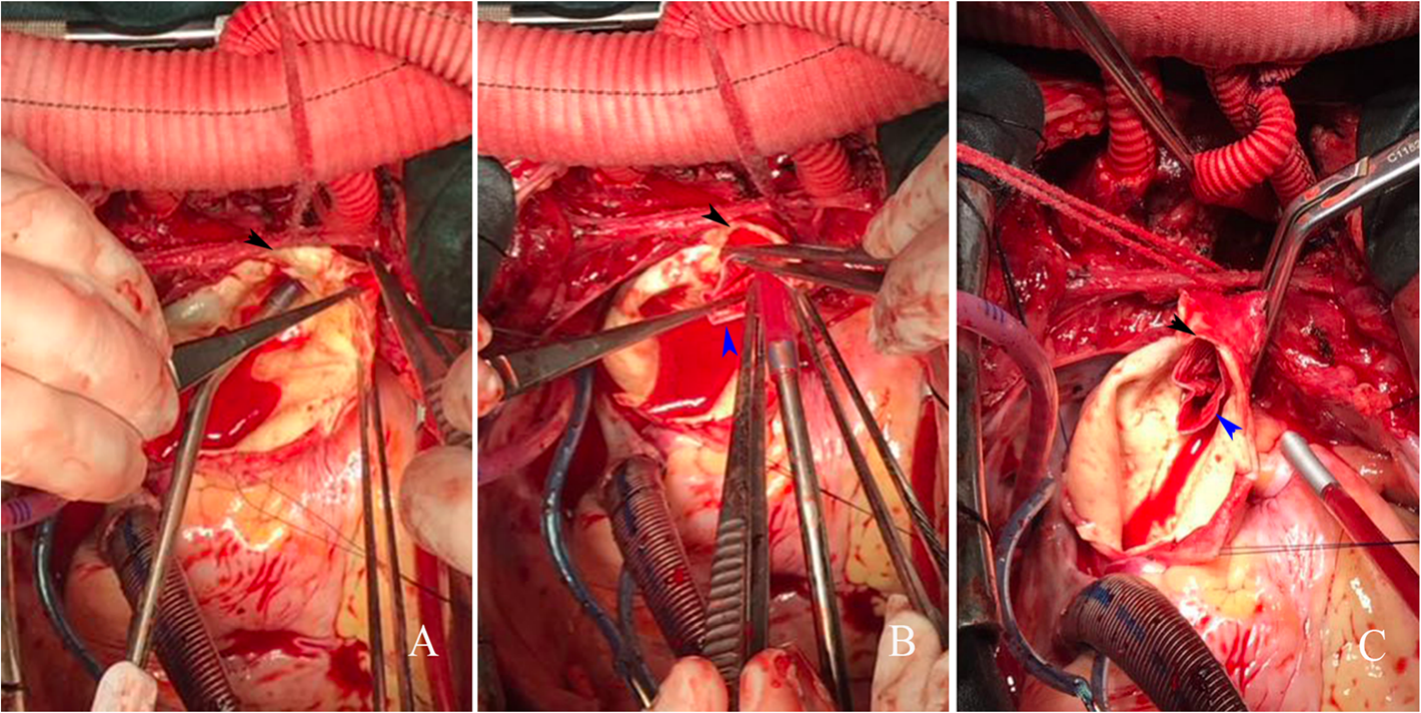
**a** Ascending aortic incision and descending aorta probing. **b** The stented graft is inserted into the descending aorta. **c** Middle arch clamping. Right brachiocephalic artery stump (black arrow); Stent-free portion of the frozen elephant stent vessel (blue arrow)

**Fig. 3:**
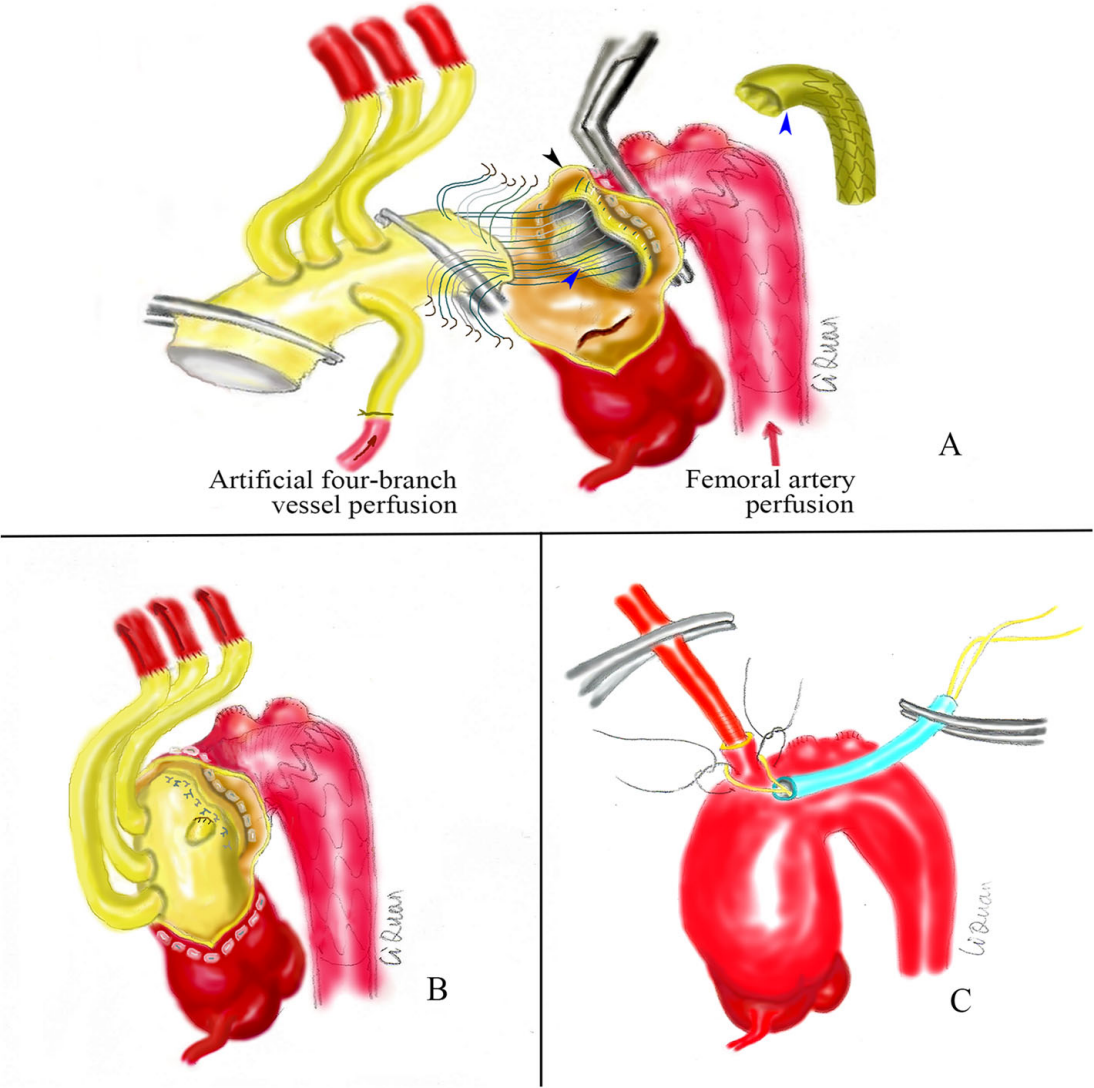
**a** Method for anastomosis of the aortic arch and four-branch vessel. Right brachiocephalic artery stump (black arrow); Stent-free portion of the frozen elephant stent vessel (blue arrow). **b** Method for anastomosis of the total four-branch vessel. **c** Cannulation of the right brachiocephalic artery stump

After the three branches were anastomosed, the ascending aorta was blocked, and full-flow extracorporeal circulation was carried out. The ascending aorta was cut longitudinally to remove the haematoma, and the root of the aorta was anterogradely infused with myocardial protection solution (Fig. 1c). Next, the extracorporeal circulation flow was reduced to 900–1300 ml/min, femoral artery perfusion was blocked, the ascending aortic cross clamp was opened, and the ascending aortic incision was extended upward to the right brachiocephalic artery stump (Fig. 2a). A 26–30 mm frozen elephant stent vessel (MicroPort Medical Co. Ltd., Shanghai, China) was implanted into the true lumen of the descending aorta (Fig. 2b). After implantation, the lumen of the stent was expanded and shaped using the left index finger. The stent-free portion of the artificial vessel was stretched to prevent the blood vessel from being twisted, and arch blocking was performed by an assistant. The middle aortic arch, including the stent-free portion of the artificial stent vessel, was blocked between the right brachiocephalic artery and the left common carotid artery (Figs. 2c and 3a). Femoral artery perfusion was restored immediately, and full-flow extracorporeal circulation was restored. At this time, the femoral artery had stopped cycling for approximately 2 min (the safe time limit for spinal cord warm ischaemia is approximately 6 min at 34 °C). First, the distal end of the artificial four-branch vessel was anastomosed with the stent-free portion of the frozen elephant incorporating the aortic arch wall. A 2–0 ETHIBOND EXCEL™ (V-5) suture was used for intermittent suturing (Fig. 3a). After the aortic sinus was remodelled, the proximal end of the ascending aorta was finally anastomosed to the proximal end of the artificial four-branch, and the suture method was consistent with that used at the distal end of the four-branch (Fig. 3b). The proximal cross clamp of the artificial blood vessel was released, and cardiac resuscitation was achieved.

No patients experienced neurological complications. One patient with combined COPD was discharged from the hospital 24 days later due to pneumonia, and the other patients were all discharged within 2 weeks. During more than 1- month of follow-up, no patients had aortic valve regurgitation or anastomotic leak.

## Discussion

The greatest advantages of using this technique of aortic “middle arch clamping” include its ability to avoid damage to the vital organs of the body during moderate or deep hypothermic circulatory arrest and significantly reduce the time of cooling and rewarming, thus reducing the operation time and chance of complications. Moreover, the anastomosis site at the distal end of the arch can be moved 3 to 4 cm in the proximal direction, making the operation more convenient. In addition, the “middle arch clamping “ technique allows more time for distal arch anastomosis, which is particularly beneficial for young surgeons who are gaining experience in arch replacement surgery. This technique requires the application of femoral artery cannula perfusion, which has its own advantages compared with axillary artery or innominate artery cannula perfusion. First, the femoral artery is easily separated; second, femoral artery cannula perfusion can ensure the supply of blood to abdominal organs and the spinal cord after middle arch clamping, while other arteries cannot.

In the application of the “middle arch clamping” technique, to ensure reliable perfusion of the femoral artery, the ascending aorta should not be cut immediately after it is blocked and the femoral artery is perfused. The tension of the blood vessels in the arch should first be palpated to roughly estimate the perfusion effect of the femoral artery. The femoral artery is considered poorly perfused if the tension in the arch is too low. The aortic cross clamp should be released immediately, and the flow rate should be reduced. The femoral artery perfusion should be blocked again, and the blood supply from the heart to the lower body should be maintained. Cannulation of the brachiocephalic artery stump should then be performed (Fig. 3c). The 12-14F arterial cannula (Fig. 1b blue arrow) should be replaced with a 20-22F aortic cannula, and traditional deep hypothermic circulatory arrest surgery should be conducted. After deep hypothermic circulatory arrest is achieved, the cannula can be removed, and the distal end of the arch anastomosed. After completion, only the fourth branch is perfused to maintain the blood supply.

The technical limitation of the “middle arch clamping” technique is that separation and blockage cannot be performed in patients with large aneurysms in the arch. For patients with severe dissection and a large pressure difference between the upper and lower limbs, femoral artery perfusion cannot guarantee blood supply to the spinal cord and internal organs. Therefore, this technique is not applicable to such patients. For these patients, we recommend the “branch-first” technique combined with “cannulation of the brachiocephalic artery stump” for perfusion (Fig. 3c) in the arch replacement procedure. The advantage of the “cannulation of the brachiocephalic artery stump” technique is that it can reduce the incisions in the axillary and femoral arteries, as well as the damage, and operation time; moreover, it can provide direct visualization of the brachiocephalic artery stump to better distinguish the true and false lumen and reduce the risk of insertion into the false lumen.

The “branch-first” technique has been described previously in a few reports [5]. The advantage of the “branch-first” technique is ensuring antegrade perfusion of the brain, thus avoiding poor perfusion of the axillary artery in patients with an incomplete cerebral arterial circle (Circle of Willis) while reducing the cardiac arrest time. This technique is mainly used in aortic arch replacement patients. Indications for arch replacement include the following: The dissection involves the aortic arch, especially an aortic intima rupture in the arch; three branches of the aorta are involved, and further dissociation may affect the blood supply to the brain.

To the best of our knowledge, in other reports, no cardiac surgeon can perform open anastomosis at the distal end of the arch with a core temperature ≥ 34 °C. The anastomosis needs to be completed at a 22 to 28 °C core temperature and at 20 to 30 min of circulatory arrest. Given these parameters, we converted open anastomosis at the distal end of the arch into closed anastomosis with continuous perfusion. Therefore, this aortic arch replacement procedure can be performed at a core temperature ≥ 34 °C and at almost continuous perfusion (only 2 min of circulatory arrest).

## Conclusions

This technique can increase the operating temperature by approximately 6 to 12 °C and reduce the circulatory arrest time by approximately 18 to 28 min. Because of the small number of cases, a definitive conclusion cannot be drawn. However, all of the patients recovered well without any neurological complications, demonstrating the feasibility and safety of this technique. We believe that this technique can serve as a good alternative strategy for managing aortic dissection and aneurysm, especially for young surgeons who are acquiring experience in arch replacement surgery.

## Data Availability

Not applicable.

## Abbreviations

COPD: Chronic obstructive pulmonary disease
CTA: Computed tomography angiography
